# Retraction Note: Neoantigen vaccine nanoformulations based on Chemically synthesized minimal mRNA (CmRNA): small molecules, big impact

**DOI:** 10.1038/s41541-025-01306-7

**Published:** 2025-11-14

**Authors:** Saber Imani, Oya Tagit, Chantal Pichon

**Affiliations:** 1 Shulan International Medical College, Zhejiang Shuren University, Hangzhou, Zhejiang China; 2 Institute of Chemistry and Bioanalytics, School of Life Sciences, University of Applied Sciences and Arts Northwestern Switzerland, Muttenz, Switzerland; 3 Center of Molecular Biophysics, CNRS, Orléans, France; 4 ART-ARNm, National Institute of Health and Medical Research (Inserm) and University of Orléans, Orléans, France; 5 Institut Universitaire de France, Paris, France

**Keywords:** Gene therapy, Translational research

Retraction to: *npj Vaccines* 10.1038/s41541-024-00807-1, published online 18 January 2024

The Editor-in-Chief has retracted this Perspective. After publication, concerns were raised regarding the accuracy of statements about Chemically synthesized minimal mRNA (CmRNA) and the references which support them. An assessment by the Journal found that these concerns are substantiated. The Editor-in-Chief therefore no longer has confidence in the soundness of this Perspective.

The authors agree with this retraction.

